# Special Issue from the NSERC Bioconversion network workshop: *pretreatment and fractionation of biomass for biorefinery/biofuels*

**DOI:** 10.1186/1754-6834-6-17

**Published:** 2013-01-28

**Authors:** Jack Saddler, Linoj Kumar

**Affiliations:** 1 Forest Products Biotechnology/Bioenergy, University of British Columbia, British Columbia, Canada

## 

World demand for energy and commodity chemicals continues to increase at a rapid pace in parallel with global industrialization and economic development. The current annual global energy demand of 13 billion tonnes of oil equivalent (btoe) is predicted to increase by 35% in 2035 [1]. The convenience, infrastructure investment and high energy content of liquid hydrocarbons makes them the preferred energy source for all modes of transportation and the dominant feedstock for the majority of today’s commodity chemicals.

However, we face significant challenges with the continued use of oil, from concerns about carbon emissions contributing to climate change to ongoing depletion of finite oil reserves affecting the lifestyles of future generations. It is inevitable that we will have to evolve from a finite, hydrocarbon driven global industry to a more sustainable, carbohydrate based society. If managed sustainably, biomass will be the major, alternative, renewable, source of many of the chemicals and fuels that we currently derive from hydrocarbons such as coal, oil and natural gas [2].

The development of sugar/starch/plant oils derived biorefineries has already shown how a range of fuels and chemicals can be produced economically and sustainably. However, in order to make a complete transition from a ‘fossil fuel economy’ to a ‘bioeconomy’, the whole barrel of oil needs to be replaced by biomass-derived counterparts [3]. Many types of chemicals and conventional fuels such as gasoline, middle distillates and aviation fuels are used today. However, as described by several of the papers in this special pretreatment issue of the journal there are several promising biomass-derived equivalents, which can potentially substitute or replace most conventional fuels and chemicals. These biomass derived molecules should be identical to those produced by oil or gas based refineries in order to be compatible with existing infrastructure and applications. The biorefinery approach considers biomass as the source of a variety of chemicals and polymers, in an analogous fashion where an oil refinery gives us the fuels and thousands of chemicals we use routinely in our daily life. In an oil refinery, liquid fuels, such as gasoline and diesel, form the bulk, relatively low value products, while the plastics, dyes and chemicals provide the higher value, “niche” markets. It is likely that a biomass based biorefinery will follow the same profile of products and values.

After collection and delivery, the first stage in any lignocellulose based biorefinery process will be the pretreatment step. In addition to enhancing the enzyme’s accessibility to cellulose, an “ideal” pretreatment will aid in the ready fractionation of the biomass into its key constituents (Cellulose, hemicellulose and lignin) while facilitating their subsequent conversion to both fuels and high value co-products in high yield and titre [3,4]. Therefore, the initial choice and type of pretreatment will be critical as it influences all subsequent downstream processing steps and the efficiency and economics of the overall process. As is described by many of the papers in this special pretreatment issue of the journal, the type of pretreatment that can be successfully used is highly dependent on the nature of the biomass feedstock and desired products that are derived from the biorefinery. However, not all biomass sources are created equal with substrates such as corn fibre considered to be relatively easy to pretreat while softwoods are typically quite recalcitrant [3,4]. It is widely recognised that the structural diversity of various biomass sources ranging from agricultural residues to hardwoods to softwoods and the need to sometimes provide a more “uniform/fine-particle” type feedstock will heavily influence the effectiveness of the various pretreatments [3,4]. An “ideal” pretreatment would be feedstock agnostic, such that it would be effective on a wide range of biomass feedstocks. The choice and method of pretreatment will also depend on the nature and the value of the co-products that could be produced from substrate components such as the lignin and extractives. Identifying and optimising a pretreatment that is “feedstock agnostic”, economical, robust and that can effectively fractionate biomass into desirable chemical components with minimum use of enzymes and chemicals continues to be a key research area [3,4] and was the focus of a recent pretreatment workshop held at the University of British Columbia (UBC).

Several years ago the Bioconversion Network was established by the Natural Science and Engineering Research Council (NSERC) of Canada to bring together several of Canada’s major researchers working in the biomass-to-fuel-and chemicals area. Realizing the primary role that pretreatment plays in accelerating the commercialisation of the overall biorefinery concept, NSERC generously supplied funds through its SNEI (Strategic Network Enhancement Initiative) program to catalyze the planning and hosting of a “pretreatment workshop” that was held over the three days of June 4-6th at the UBC Forest Sciences Centre, Vancouver, British Columbia. There were 146 active participants including representatives from industry (62), academia and government (42) while the graduate students and post-docs (38) in attendance emphasised the training goal of the workshop. The meeting brought together leaders in pretreatment/bioconversion research from forest products/biorefinery/biotechnology companies such as Catchlight, Iogen, Abengoa, Novozymes, Green fields, Lignol, Andritz, Metso, Mascoma, Advanced Bio, FP Innovations, Alpac and Alberta Innovates, etc., as well as many of the recognised **world experts in biomass pretreatment**.

Many of the pretreatment technologies that are being actively investigated and commercialised around the world were described and discussed. These ranged from processes that are closer to commercialisation such as variations on steam/dilute acid pretreatments [5] that are being pursued by companies such as Inbicon, Abengoa and Andritz, to variations of “traditional” pulping processes, to more “evolving” processes such as the potential of ionic liquid. To facilitate discussion, interaction and a more workshop *modus operandi,* the meeting was organised into distinct sessions including scale-up challenges, commercialization, reactor systems technology, techno-economics and biomass energy supply chains and strategies for integrated biorefining. Within these sessions, the group discussed what lessons could be learned from related industries such as the oil refinery, pulp and paper and corn ethanol industries. There was an emphasis on how the global, and Canadian forest products industry in particular, could expand its current product portfolio into more of a biorefinery concept. The workshop also highlighted the potential opportunities and challenges of sourcing biomass, and the related logistics issues. The panel session entitled “Substrate characterization and fundamentals of pretreatment” covered a range of advanced tools/techniques to try to better understand the structural changes occurring in biomass during pretreatment and the subsequent influence on enzymatic hydrolysis, sugar fermentation and coproduct production. The workshop concluded with a panel entitled, “Impressions, priorities and the future of pretreatment” that attempted to “look-into-the-crystal ball” to forecast the future needs of pretreatment on both the industrial and more fundamental fronts. There was a consensus that, “there is no clear “ideal pretreatment” and that it is unlikely that one single pretreatment will be universally used”. Rather, several different pretreatment processes and combination will be employed to derive both the high volume/lower value sugar-based products and the lower volume/higher value niche markets. However, it was also agreed that obtaining high yields of “clean” monomeric sugars at high concentration using low enzyme/chemical input will continue to be a key goal for any pretreatment as these sugars can be transformed into both fuels and building-block chemicals by fermentation as well as by enzymatic and chemical transformations.

The workshop proved to be a unique forum by bringing together many of the individuals and companies working in the traditional and evolving “refinery” sectors. It also benefited from an animated dialogue between world renowned international experts in biomass pretreatment, oil, pulp and paper, enzyme, ethanol and biotechnology companies, as well as equipment manufacturers and end users. The insightful questions of the next generation of engineers, scientists and policy makers that constitute the graduate students and Post docs in attendance also added to the success of the meeting. Presenters were invited to try and capture the insights they provided at the workshop and make this information available to a wider audience by publication in a leading open access journal. This special “Pretreatment” Issue of *Biotechnology for Biofuels* brings together some of the peer reviewed articles, which were presented and discussed at the workshop. We want to thank all of our colleagues who made the workshop a success by not only taking time out of their busy schedules but also by their openness, generosity with their experience and the depth of their insights. We hope that this special “pretreatment” issue will help update the bioconversion/biorefinery research community on some of the advances in pretreatment research, development and demonstration (R, D&D).

We want to thank the Editors of the Journal “*Biotechnology for Biofuels*” and Dr Helen Whitaker and her colleagues at BioMed Central for their help in the editorial process. We also want to thank the many colleagues who helped in the peer review process, strengthening the quality of the manuscripts, and all of the contributors to the special issue.
